# Biotransformation of Bisphenol AF to Its Major Glucuronide Metabolite Reduces Estrogenic Activity

**DOI:** 10.1371/journal.pone.0083170

**Published:** 2013-12-13

**Authors:** Ming Li, Yunjia Yang, Yi Yang, Jie Yin, Jing Zhang, Yixing Feng, Bing Shao

**Affiliations:** 1 Beijing Key Laboratory of Diagnostic and Traceability Technologies for Food Poisoning, Beijing Center for Disease Control and Prevention, Beijing, China; 2 School of Public Health and Family Medicine, Capital Medical University, Beijing, China; Northwest Fisheries Science Center, NOAA Fisheries, United States of America

## Abstract

Bisphenol AF (BPAF), an endocrine disrupting chemical, can induce estrogenic activity through binding to estrogen receptor (ER). However, the metabolism of BPAF *in vivo* and the estrogenic activity of its metabolites remain unknown. In the present study, we identified four metabolites including BPAF diglucuronide, BPAF glucuronide (BPAF-G), BPAF glucuronide dehydrated and BPAF sulfate in the urine of Sprague-Dawley (SD) rats. BPAF-G was further characterized by nuclear magnetic resonance (NMR). After treatment with a single dose of BPAF, BPAF was metabolized rapidly to BPAF-G, as detected in the plasma of SD rats. Biotransformation of BPAF to BPAF-G was confirmed with human liver microsomes (HLM), and V_max_ of glucuronidation for HLM was 11.6 nmol/min/mg. We also found that BPAF glucuronidation could be mediated through several human recombinant UDP-glucuronosyltransferases (UGTs) including UGT1A1, UGT1A3, UGT1A8, UGT1A9, UGT2B4, UGT2B7, UGT2B15 and UGT2B17, among which UGT2B7 showed the highest efficiency of glucuronidation. To explain the biological function of BPAF biotransformation, the estrogenic activities of BPAF and BPAF-G were evaluated in ER-positive breast cancer T47D and MCF7 cells. BPAF significantly stimulates ER-regulated gene expression and cell proliferation at the dose of 100 nM and 1 μM in breast cancer cells. However, BPAF-G did not show any induction of estrogenic activity at the same dosages, implying that formation of BPAF-G is a potential host defense mechanism against BPAF. Based on our study, biotransformation of BPAF to BPAF-G can eliminate BPAF-induced estrogenic activity, which is therefore considered as reducing the potential threat to human beings.

## Introduction

With a similar structure to the synthetic estrogen bisphenol A (BPA), bisphenol AF (4,4´-hexafluoroisopropylidene-2-diphenol, BPAF) is used primarily as a monomer for polyimides, polyamides, polyesters and other specialty polymers and as a cross linker for certain fluoroelastomers [1,2]. In 2008, BPAF was nominated by the National Institute of Environmental Health Sciences (NIEHS) for comprehensive toxicological characterization based on its moderate production [1]. The presence of BPAF was reported in the environmental samples collected around a manufacturing plant which is one of the largest BPAF manufacturers in China [3]. It has been well-documented that BPAF could bind strongly to estrogen receptor (ER) *in vitro*, and function as an endocrine disrupting chemical (EDC) by acting as an agonist for ERα and antagonist of ERβ to mediate physiological processes [4-6]. BPAF also could activate the human pregnane X receptor, a nuclear receptor that functions as a regulator of xenobiotic metabolism [7]. Our recent study demonstrated that BPAF can cause reduction of testosterone by directly affecting testis function in adult male rats [8]. 

UDP-glucuronosyltransferases (UGTs) that were constitutively expressed in a tissue-specific manner play a key role in the glucuronide biotransformation of xenobiotics and endogenous substances [9-11]. It has been reported that UGTs were closely associated with the metabolism and toxicity of BPA in mammals [12,13]. The major metabolite of BPA is the monoglucuronide conjugate in the urine and plasma of rats [13]. In the *in vitro* metabolism studies, BPA could be metabolized to BPA glucuronide by UGT2B1 in rat liver microsomes [14,15] and by human recombinant UGT isoforms [11]. Moreover, BPA also could be metabolized to 3-hydroxy BPA and BPA o-quinone by cytochrome P450s *in vitro* [16,17]. Recently, Schmidt et al reported that P450 could mediate biotransformation of BPAF to hydroxylated BPAF, followed by the central carbon bridge degradation which product 4-hexafluorohydroxyisopropylidene-phenol as the main metabolite in the presence of human liver microsomes (HLM) with NADPH and GSH *in vitro* [18]. However, the biotransformation of BPAF *in vivo* and the estrogenic effect of its metabolites remain unknown.

The information on potential toxicities, metabolism, environmental presence and environmental fate of BPAF is limited. It is important to understand BPAF’s biotransformation to better estimate the potential threat to human beings. Therefore, our aim is to identify and characterize the metabolites of BPAF both *in vivo* and *in vitro*. Moreover, we evaluate the estrogenic activities of BPAF and its metabolites to explain the biological function of BPAF biotransformation *in vivo*. 

## Materials and Methods

### Chemicals and reagents

BPAF (98% purity) was purchased from Tokyo Chemical Industry Co., Ltd. (Tokyo, Japan); HPLC grade acetonitrile and methanol (MeOH) were purchased from Fisher Scientific (Fair Lawn, NJ, USA); and corn oil and DMSO-d_6_ was obtained from Sigma Aldrich (St. Louis, MO, USA). UGT reaction mix solution A, UGT reaction mix solution B, NADPH reaction buffer, pooled HLM and human recombinant UGTs (UGT1A1, 1A3, 1A4, 1A6, 1A7, 1A8, 1A9, 1A10, 2B4, 2B7, 2B15 and 2B17) were obtained from BD Biosciences (San Jose, CA, USA).

### Animal studies

Male Sprague-Dawley (SD) rats 8 weeks of age (250-300 g) were purchased from the experimental animal center of the Academy of Military Medical Sciences, China. Animals were housed in steel metabolism cages for acclimation 3 days before the experiment. All rats were allowed free access to standard rat chow and water. Due to the limited solubility in saline solution, BPAF was dissolved in corn oil at the concentration of 100 mg/mL for animal experiments. A dose of 200 mg/kg BPAF was administered daily by oral gavage for 2 consecutive weeks. Total urine samples were collected and pooled everyday, and samples were chilled on ice. 

For evaluating the metabolites of BPAF in plasma, 6 rats randomly divided into two dose groups were orally administrated by a single dose of 20 mg/kg or 100 mg/kg BPAF. 100 µL blood samples were collected at 0.5, 1, 2, 3, 6, 12, 24 and 48 h in heparinized tubes by direct puncture of the tail vein, chilled immediately on ice and centrifuged for 10 min at 3,000 g at 4 °C. The resulting plasma was stored at -20 °C until analysis. All of the experiments procedures using animals were approved by Animal Care and Use Committee of Beijing Center for Disease Control and Prevention.

### Isolation and Purification of BPAF metabolites

Because of the lack of commercial standards for BPAF metabolites, four metabolites were isolated and purified from the urine of SD rats by semi-preparative HPLC as described in detail in Method S1 in File S1.

### UPLC/ESI-QTOF-MS analysis

Metabolites of BPAF were identified by ultra-performance liquid chromatography/electrospray ionization quadrupole time-of-ﬂight mass spectrometry (UPLC/ESI-QTOF-MS). Frozen urine samples were thawed, vortex-mixed, then centrifuged at 7,000 g for 10 min. 5 μL raw urine was directly injected into the UPLC. Liquid chromatographic separations were conducted using a Waters Acquity UPLC^TM^ system (Milford, MA, USA) with a BEH C18 column (2.1 mm ×100 mm; particle size, 1.7 µm) from Waters (Milford, MA, USA). The UPLC conditions for separating metabolites are described in Method S2 in File S1.

The MS^E^ analysis, which provided information on the intact and fragment ions of products and metabolites in a single sample injection, was performed on a Waters Synapt^TM^ Q-TOF mass spectrometer (Milford, MA, USA) under negative electrospray ionization condition in V-mode. The capillary, sampling cone and extraction cone voltages were set to 3.1 kV, 35 V and 4 V, respectively. Source and desolvation temperatures were held at 110 °C and 400 °C, respectively. The cone gas and desolvation gas used was nitrogen, and they were set at 50 L/h and 800 L/h, respectively. Their functions were run for data acquisition in centroid mode with *m/z* 50-1,000. For MS scan, trap collision energy was set to 6.0 eV, 20 eV, and 30 eV. An external reference solution containing 1 mg/L of leucine enkephalin (*m/z* 554.2615) was used for mass lock. 

### UPLC/ESI-MS/MS analysis

The quantification of BPAF and BPAF-G was conducted by ultra-high-pressure liquid chromatography/electrospray ionization tandem mass spectrometry (UPLC/ESI-MS/MS) in negative ionization mode. 400 μL acetonitrile was added to 100 μL plasma sample. The mixture was sonicated at room temperature for 15 min, then centrifuged at 7,000 g for 10 min to precipitate proteins. The supernatant was dried under a gentle stream of nitrogen, and the residual was reconstituted with 500 μL MeOH/H_2_O (50/50, v/v) for UPLC/ESI-MS/MS analysis. Liquid chromatographic separations were performed using a Waters Acquity UPLC^TM^ system (Milford, MA, USA) with a BEH C18 column (2.1 mm ×50 mm; particle size, 1.7 µm) from Waters (Milford, MA, USA). The mobile phase was solvents A (methanol) and B (water). With a flow rate of 0.4 mL/min, gradient elution was operated with 20% A, followed by a 4 min linear gradient to 100% A and kept for 2 min. The system was re-equilibrated for 3 min between runs. The MS used was a Xevo triple quadrupole mass spectrometer (Milford, MA, USA). The capillary voltage and source temperature were set at 2.7 kV and 150 °C, respectively. The desolvation temperature and nitrogen flow rate were set at 400 °C and 1,000 L/h, respectively. Argon was used as the collision gas at a flow rate of 0.16 mL/min. The MS/MS acquisition parameters were optimized in ESI negative mode for maximum sensitivity. The quantification of BPAF and BPAF-G was performed by Multiple Reaction Monitoring (MRM) mode, MRM transitions and collision energies (Ecoll) for quantification were 335.2 > 265.0 Ecoll = 25 eV for BPAF, 510.8 > 112.9 Ecoll = 20 eV for BPAF-G; MRM transition and Ecoll for conformation were 335.2 >197.0 Ecoll = 35 eV for BPAF, 510.8 > 265.0 Ecoll = 40 eV for BPAF-G. Quantification of BPAF and BPAF-G was based on six-point matrix-fortified curves obtained after addition of known amounts of BPAF and BPAF-G to blank plasma samples from untreated control rats. Calibration curves were linear from 1 µg/L to 250 µg/L for BPAF and from 10 µg/L to 2500 µg/L for BPAF-G. The correlation coefficients (*r* ^2^) of the matrix-fortified calibration curves were all greater than 0.99. Method accuracy evaluated by spiked recovery at three levels (*n* = 5) were ranged from 82.8% to 106.7%, with precision (RSD %) no more than 10.9%. The method limits of quantization (MLOQ) of BPAF and BPAF-G in the plasma were 1 µg/L and 10 µg/L, respectively. More details were described and summarized in Method S3 and Table S1 in File S1.

### In vitro metabolism

UGT activity assay followed the general procedure in the data sheets provided by BD Biosciences with minor modifications. Briefly, 10 µL pooled HLM (5 mg/mL) or individual human recombinant UGT (5 mg/mL) was pre-incubated with 100 µL UGT reaction mix solution B and 340 µL purified water at 37°C for 5 min in a dry bath incubator. Reactions were initiated by the addition of substrates containing 40 µL UGT reaction mix solution A and 10 µL BPAF (500 µM). V_max_ represents the maximum velocity achieved by UGT reaction system at the saturating concentrations of all substrates. For hydrolysis assay, 2 µL BPAF (5 mM) was incubating with 15 µL HLM (5 mg/mL) in the presence of NADPH systems, including 713 µL purified water, 200 µL potassium phosphate (0.5 M and PH 7.4), 50 µL NADPH regenerating solution A and 20 µL NADPH regenerating solution B. Reactions were stopped by adding equal volume of acetonitrile to the reaction mixture. After centrifugation at 10,000 g for 3 min, supernatant samples after 1,000-fold dilution by MeOH/H_2_O (50:50, v/v) were analyzed as described above.

### Cell culture

T47D and MCF7 cells were purchased from Cell Bank (Shanghai Institute of Biochemistry and Cell Biology, Chinese Academy of Sciences) and cultured in RPMI-1640 medium supplemented with 10% (v/v) fetal bovine serum (FBS) (Gibco) at 37°C and 5% CO_2_/95% air (v/v). At 70% confluence, the medium was changed to RPMI-1640 medium supplemented with 10% charcoal-stripped fetal bovine serum (SERANA, Australia) for 3 days before treatment in order to minimize the estrogen activity from FBS. BPAF was dissolved in DMSO at a concentration of 1 M as a stock solution, and working solutions were prepared freshly by diluting the stock in cell culture medium.

### RNA isolation and real-time PCR analysis

Total RNA from breast cancer cells was extracted using the RNeasy Mini Kit in accordance with the manufacturer’s protocol (QIAGEN). 1 µg total RNA was reverse-transcribed using using Superscript reverse transcriptase (Promega). Primers for Cathepsin D (CTSD) were 5’-CAGCCAGGCATCACCTTCAT-3’ and 5’-CAGGTAGAAGGAGAAGATGT-3’, primers for growth regulation by estrogen in breast cancer 1 (GREB1) were 5’-ATTGGTGGACCGATTGCTCA-3’ and 5’-GCTGATGAGGGTGTGCTGTGT-3’, primers for trefoil factor 1 (TFF1) were 5’-GTGCAAATAAGGGCTGCTGTT-3’ and 5’-CACACTCCTCTTCTGGAGGGA-3’ and primers for GAPDH were 5’-CACCCACTCCTCCACCTTTGA-3’ and 5’-ACCACCCTGTTGCTGTAGCCA-3’. The reactions were performed using Power SYBR Green PCR Master Mix (Applied Biosystems) in an ABI Prism 7300 Sequence Detection system for 40 cycles (95 °C 15 sec, 60 °C for 60 sec) after an initial 10 min incubation at 95 °C. The fold change in the expression of each gene was calculated using 2^-ΔΔCt^ method.

### Cell proliferation assay

Cell proliferation was determined by the WST-1 dye (#C0038 Beyotime, China). Briefly, T47D and MCF7 cells were seeded in 96-well plate at the concentration of 3,000 cells per well. After treatment with BPAF or BPAF-G for 7 days, cell proliferation was determined using WST-1 according to the manufacturer’s instruction. 

### Statistical analysis

Statistical analysis was performed using one-way ANOVA followed by a Tukey-Kramer multiple comparison test. A *p* value < 0.05 was considered statistically significant. 

## Results

### Identification and characterization of the metabolites of BPAF in SD rats

**Figure 1:**
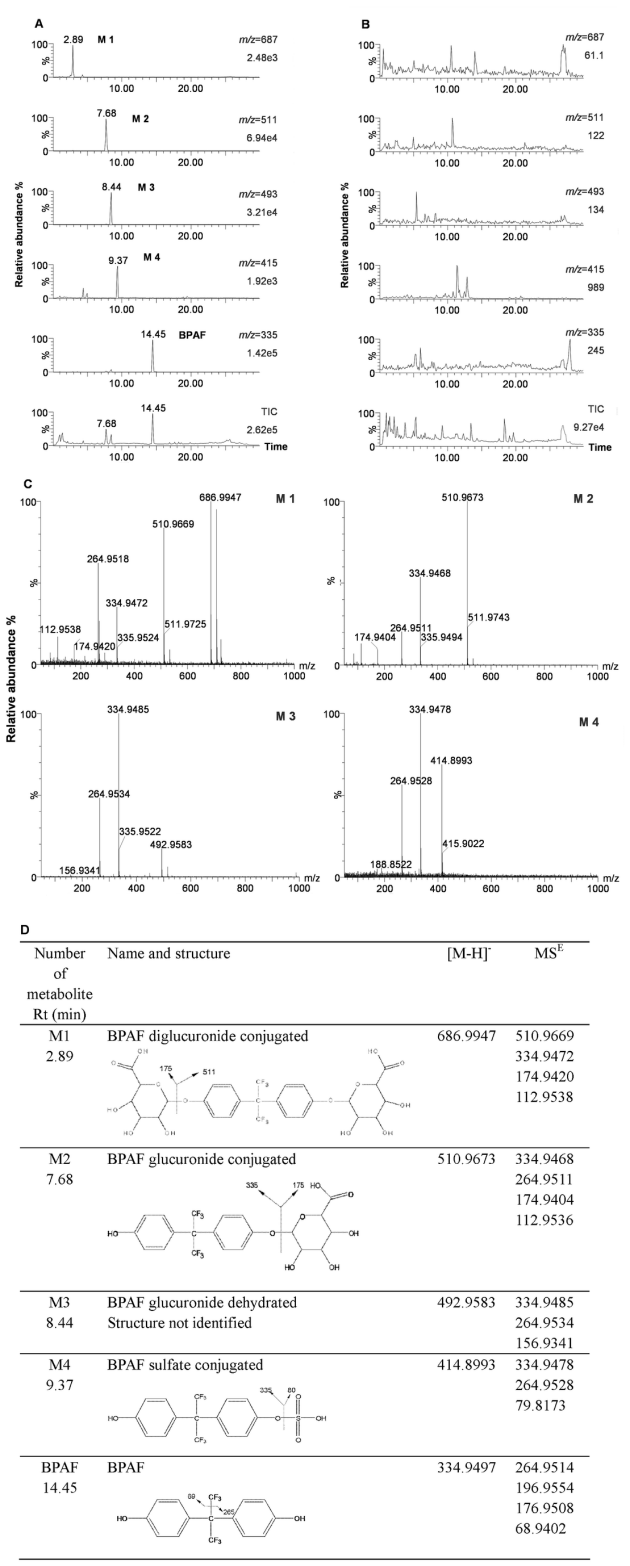
Identification of the metabolites of BPAF *in* *vivo*. SD rats were orally administrated with 200 mg/kg/day of BPAF for 2 weeks. We analyzed the metabolites of BPAF in the urine of BPAF-treated SD rats (A) and control rats (B) by UPLC/ESI-QTOF-MS. TIC, total ion chromatogram. (C) UPLC/ESI-QTOF-MS^E^ electrospray spectra of BPAF diglucuronide conjugation (M1), BPAF glucuronide (M2), BPAF glucuronide dehydrated (M3) and BPAF sulfate (M4) in the urine of BPAF-treated SD rats. (D) Mass spectrometric data and structure of BPAF metabolites and BPAF.

To identify BPAF metabolites, we performed full-scan UPLC/ESI-QTOF-MS to analyze the possible metabolites by comparing the chromatogram and mass spectrum data of BPAF-treated urine samples (Figure 1A) with those of untreated control samples (Figure 1B). Four metabolites with the retention times of 2.89 min, 7.68 min, 8.44 min and 9.37 min, marked as M1, M2, M3 and M4, were identified in the urine of BPAF-treated SD rats. The electrospray spectra of the four metabolites were analyzed by the MS^E^ mode of UPLC/ESI-QTOF-MS (Figure 1C). The most polar metabolite, M1, yielded a *m/z* 686.9947, corresponding to the diglucuronide of BPAF ([M−H] ^−^). The fragmentation of this ion led to the formation of ions at *m/z* 510.9669 and *m/z* 334.9472, resulting from the cleavage of either one or two glucuronic acid moiety from the precursor ion. Thus, this metabolite was identified as BPAF diglucuronide. The metabolite M2 gave an [M−H] ^−^ ion at *m/z* 510.9673. The fragmentation of this ion resulted in the loss of the glucuronic acid moiety and led to the formation of the *m/z* 334.9468. The fragment *m/z* 264.9511 corresponded to the loss of CF_3_ from the BPAF moiety. Fragments *m/z* 174.9404 and *m/z* 112.9536 corresponded to the glucuronic acid and the loss of CO_2_ and H_2_O from glucuronic acid moiety. Thus, the metabolite characterized as a glucuronide conjugation of BPAF was considered as BPAF glucuronide (BPAF-G), which produced the strongest responses among the four metabolites in the UPLC/ESI-QTOF-MS. The metabolite M3 had an [M−H] ^−^ ion at *m/z* 492.9583 with a retention time of 8.44 min and generated similar fragments to M2. The only different ion was found at *m/z* 156.9341, corresponding to the loss of H_2_O from the glucuronic acid moiety. M3 was characterized as BPAF glucuronide dehydrated, but its structure could not be determined because of the uncertainty of the dehydration position on the glucuronic acid ring. The precursor ion of M4 was [M−H] ^−^ at *m/z* 414.8993. The product ions *m/z* 334.9478 and 79.8173 corresponded to the BPAF moiety and sulfuric acid, enabling the precursor ion to be identified as the sulfate conjugation of BPAF. The data and structure of four metabolites and BPAF analyzed by mass spectrometry are summarized in Figure 1D.

Because standards of BPAF metabolites were not commercially available, we isolated and purified the metabolites from the urine of SD rats to further confirm the structural characterization by nuclear magnetic resonance (NMR). Due to the limited amount of M1 in the urine, amounts of M2, M3 and M4 components were sufficient for NMR analysis. However, M3 and M4 components collected with poor purity could not be applied for NMR. Only the M2 component was further characterized by ^1^H and ^13^C NMR (Figure S1 in File S1). In the ^1^H and ^13^C NMR spectra, the resonances for carbons and protons of the aglycone moiety had a close resemblance to those of BPAF [19,20]. In addition, the characteristic ^1^H and ^13^C NMR data of glucuronic acid with signals for an anomeric proton (δ5.06, 1H, d) and a carboxylic group at C-6″ at δ171.0, were present. The full assignments of carbon and proton signals are summarized in Table S2 in File S1. Our data finally verified that M2 component was BPAF-4-O-glucuronide. 

**Figure 2:**
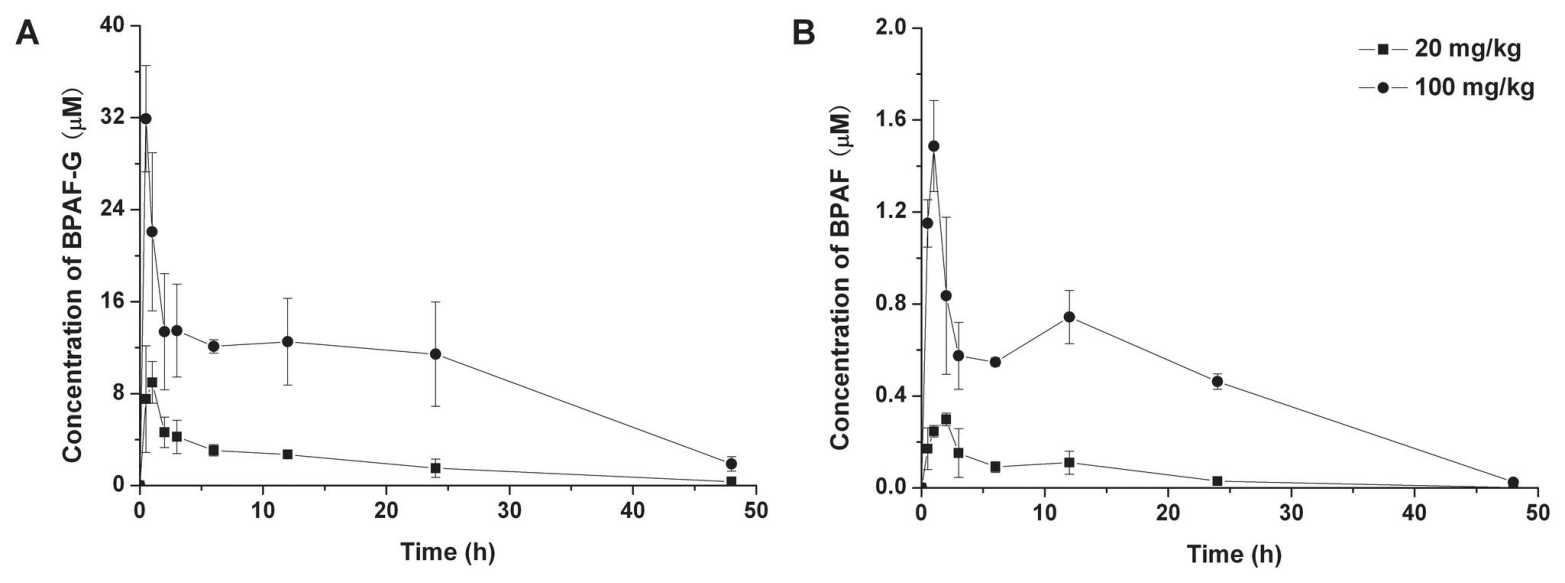
Biotransformation of BPAF in the plasma of SD rats. (A) The concentrations of BPAF-G in the plasma. (B) The concentrations of BPAF in the plasma. A single dose of 20 mg/kg or 100 mg/kg of BPAF was administered orally to SD rats, the concentrations of BPAF and BPAF-G in the plasma were detected by UPLC/ESI-MS/MS at the indicated time point. Data represent mean ± SD, *n* = 3.

To further evaluate the biotransformation of BPAF *in vivo*, we also detected the concentrations of BPAF and BPAF-G in the plasma of SD rats using UPLC/ESI-MS/MS. After treatment with a single dose of 20 mg/kg or 100 mg/kg BPAF, the peak of BPAF-G in plasma was observed at 30 min followed by rapid decline in the next three hours, representing quick clearance of BPAF in the rats (Figure 2A). However, the peak of BPAF was achieved at 1 h and eliminated completely at 48 h (Figure 2B). We also found a non-significant decline of BPAF-G concentration from the 6 h to 24 h and the second peak of BPAF at 12 h, which may be as a result of enterohepatic circulation. Moreover, we observed that the maximum concentration of BPAF-G is about 30-40-fold more than that of BPAF. Our data indicated that biotransformation of BPAF to BPAF-G occurred rapidly *in vivo*. However, M1, M3 and M4 were not observed in the plasma of rats exposed with 100 mg/kg BPAF. Moreover, after isolation and purification of the four metabolites from the urine, M2 was the only metabolite available for NMR analysis indicating its high abundance in the urine. Taken together, BPAF-G is therefore considered as the major metabolite *in vivo*.

### Biotransformation of BPAF *in vitro*

Full-scan UPLC/ESI-QTOF-MS was performed to check the possible metabolites of BPAF in the incubation with HLM. BPAF-G was the only metabolite identified after incubation for 90 min (Figure S2 in File S1). Then, we quantified the concentrations of BPAF and BPAF-G in the glucuronidation reaction system using UPLC/ESI-MS/MS. We observed that the amount of BPAF decrease is equal to that of BPAF-G increase for the indicated time (Table 1), suggesting that BPAF is transformed completely to BPAF-G by HLM in the presence of UGT reaction buffer. The V_max_ of BPAF glucuronidation for HLM was 11.6 nmol/min/mg of microsomal protein. 

**Table 1:** The concentrations of BPAF and BPAF-G in the incubation with HLM.

Time (min)	BPAF (µM)	BPAF-G (µM)
0	9.9 ± 0.26	0
1	8.80 ± 0.32	1.22 ± 0.08
2	7.79 ± 0.25	2.34 ± 0.15
5	4.21 ± 0.34	5.66 ± 0.40
10	1.21 ± 0.10	8.72 ± 0.69
15	0.91 ± 0.03	9.37 ± 0.61
20	0.31 ± 0.01	9.52 ± 0.84
30	0.11 ± 0.01	9.89 ± 0.84

Data represent mean ± SD, *n* = 3

**Figure 3:**
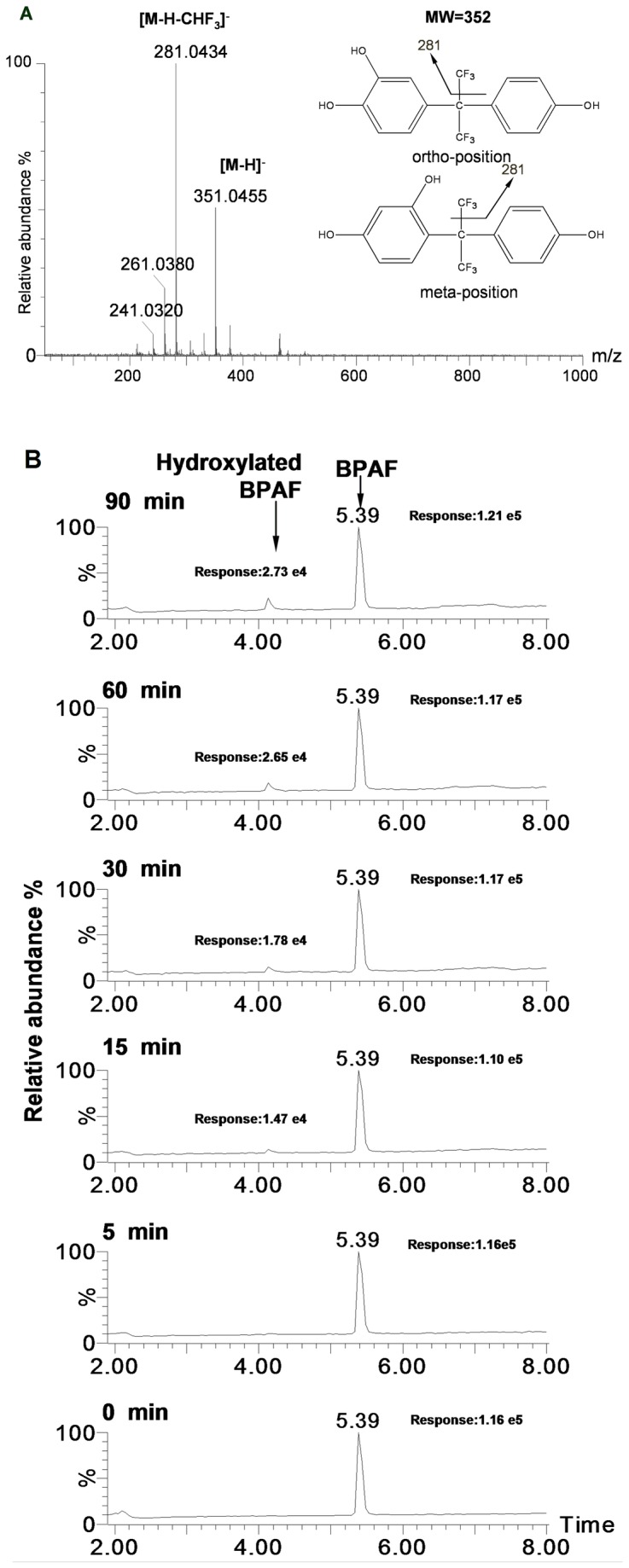
Biotransformation BPAF to hydroxylated BPAF by HLM. (A) Identification of hydroxylated BPAF. Typical mass spectrum of BPAF metabolite that yielded in the presence of NADPH was identified as hydroxylated BPAF. (B) Chromatograms of BPAF and hydroxylated BPAF at indicated time. BPAF (10 µM) was incubated with HLM (100 µg/mL) in the presence of NADPH reaction buffer, the samples were collected at indicated time points from the reaction mixture, both BPAF and hydroxylated BPAF were detected by UPLC/ESI-QTOF-MS.

In addition, we observed that HLM could catalyze the formation of hydroxylated BPAF in the presence of NADPH (Figure 3A), which is consistent with Schmidt et al’s report [18]. However, the rate of hydroxylation is obviously slower than that of glucuronidation, because the response value of BPAF did not change substantially from 0 to 90 min (Figure 3B). These results suggest that glucuronidation was more efficient than hydroxylation for BPAF biotransformation. 

**Figure 4:**
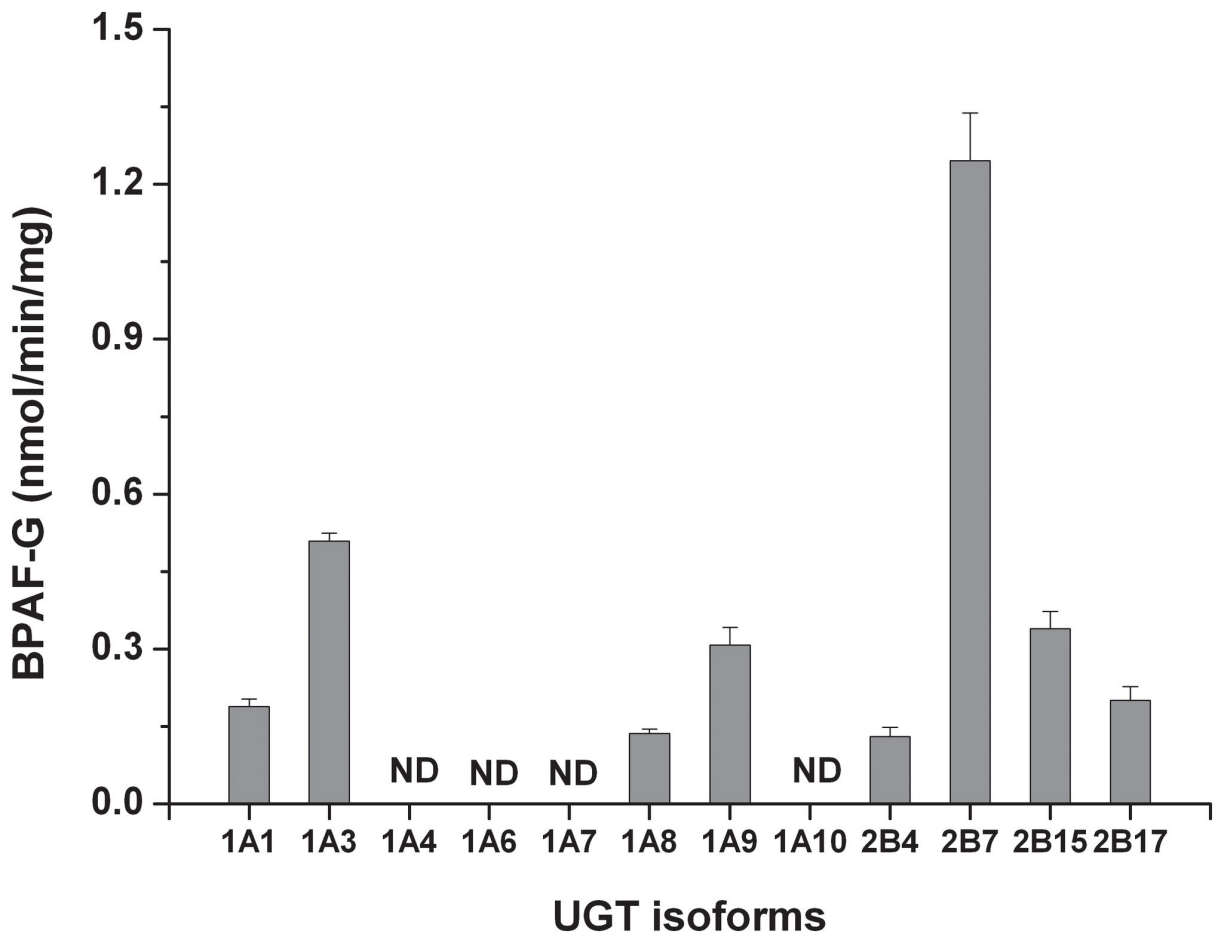
Biotransformation of BPAF to BPAF-G by human recombinant UGTs. BPAF (10 µM) was incubated with 12 individual human recombinant UGT and UGT reaction buffer, the samples were collected at 20 min and detected by UPLC/ESI-MS/MS. Data present mean ± SD of three independent experiments. ND = not detected.

Next, we also investigated whether glucuronidation of BPAF is regulated by UGT isoforms, which is the specific enzyme family responsible for the process of glucuronidation [21]. As shown in Figure 4, several UGTs were capable of mediating BPAF biotransformation, and the glucuronidation efficiency of UGTs for BPAF is UGT2B7 > UGT1A3 > UGT2B15 and UGT1A9 > UGT2B17 and UGT1A1 > UGT1A8 and UGT2B4. However, we did not detect any generation of BPAF-G in the reaction mix with the other UGT isoforms including UGT1A4, UGT1A6, UGT1A7 and UGT1A10. The capability of UGTs examined to catalyze BPAF glucuronidation (V_max_ of UGT2B7 is 1.25 nmol/min/mg) is over 9 times lower than that of HLM, suggesting that other enzymes existed in HLM could facilitate BPAF glucuronidation.

### Estrogenic activities of BPAF and BPAF-G

**Figure 5:**
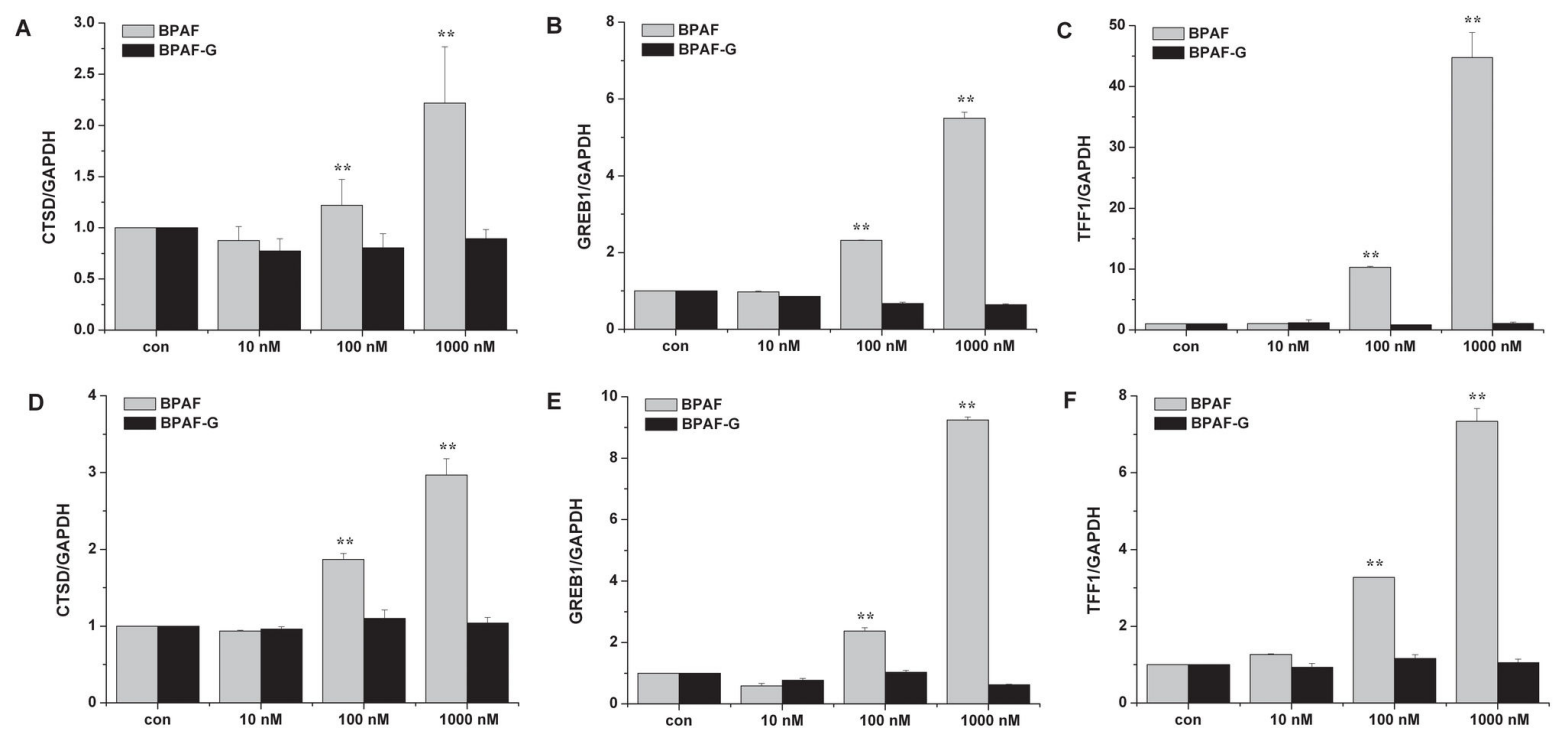
Effects of BPAF and BPAF-G on gene expression in T47D and MCF7 cells. mRNA levels of CTSD (A, D), *GREB1* (B, E) and TFF1 (C, F) were measured by real-time PCR in T47D (A, B, C) and MCF7 (D, E, F) cells after treatment with different concentrations of BPAF and BPAF-G for 24 h. Data present mean ± SD of three independent experiments. ** *p* < 0.01 compared with control.

**Figure 6:**
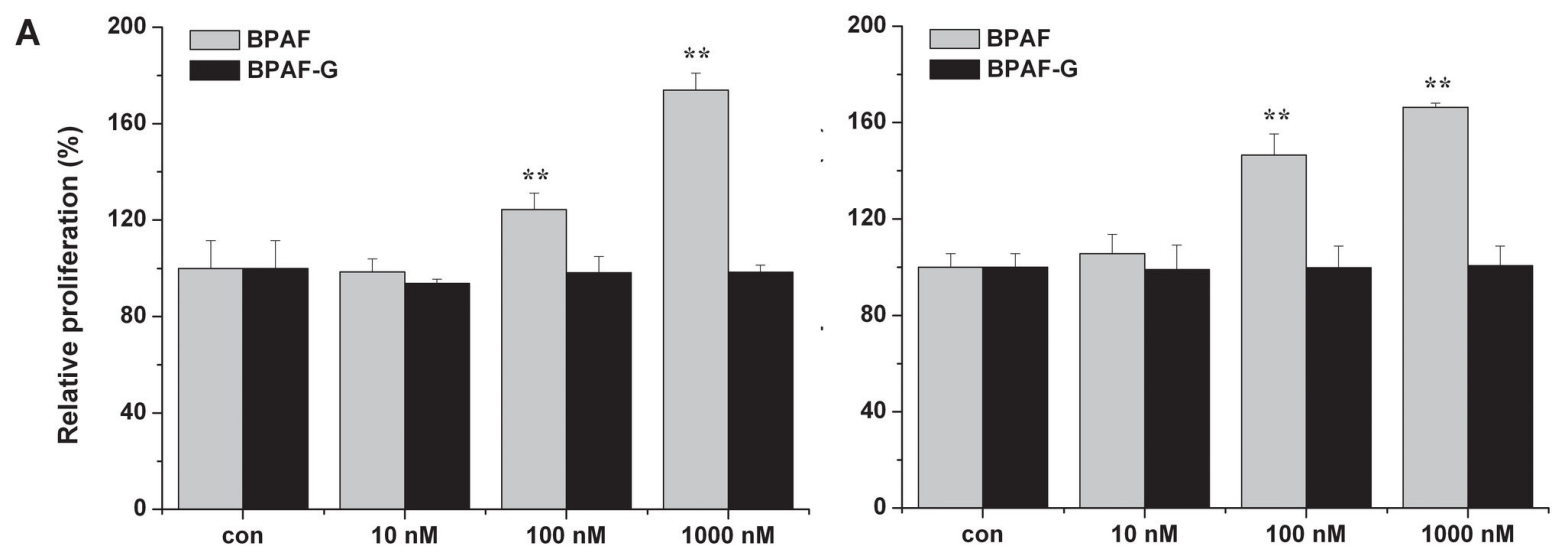
Effects of BPAF and BPAF-G on the proliferation in T47D and MCF7 cells. Proliferation of T47D (A) and MCF7 (B) cells was measured at day 7 after treatment with different concentrations of BPAF and BPAF-G. Data present mean ± SD of three independent experiments. ** *p* < 0.01 compared with control.

To determine whether biotransformation could modulate estrogenic activity of BPAF, we evaluated the estrogenic activities of BPAF and its major metabolite BPAF-G via gene expression (Figure 5) and cell proliferation (Figure 6) in ER-positive breast cancer T47D and MCF7 cells. Regulation of estrogenic activities is a multifactorial and complex process, involving both genomic and nongenomic actions. In the classical genomic action, ERα-regulated transcription occurs in a ligand-dependent manner that ERα binds to the estrogen responsive elements (EREs) within the promoter proximal regions of known estrogen-responsive genes, such as *CTSD*, *GREB1* and *TFF1* [22]. Expression of the lysosomal protease *CTSD* is induced by ERα via proximal promoter region and distal ERE binding sites located at 9 and 33 kb upstream of the transcription start site [22]. Regulation of *GREB1* expression, which plays a role in estrogen-induced proliferation of breast cancer cells, is mediated by binding of ERα to three consensus EREs spread over approximately 20 kb of upstream flanking sequences of *GREB1* [23]. Transcription of *TFF1* is commonly used to evaluate the estrogenic activity in human breast cancers [5]. In the present study, expression of ER-regulated genes was measured by real-time PCR. BPAF significantly induced expression of *CTSD*, *GREB1* and *TFF1* genes in a dose-dependent manner at concentrations of 100 nM to 1 μM in both T47D (Figure 5A-C) and MCF7 (Figure 5D-F) cells. In contrast, BPAF-G did not stimulate ER-regulated gene expression compared to BPAF. In consistence with gene expression, proliferations of T47D (Figure 6A) and MCF7 (Figure 6B) cells were stimulated by BPAF at the dose of 100 nM and 1 μM, but not by BPAF-G at the same doses. It is believed that BPAF-G does not show any estrogenic activity in ER-positive breast cancer cells. Therefore, biotransformation of BPAF to BPAF-G completely eliminated BPAF-induced estrogenic activities, which implies that formation of BPAF-G is a potential defense mechanism against BPAF toxicity *in vivo*. 

## Discussion

Increasing evidence shows that exposure to BPAF can cause adverse effects both in animal models and *in vitro* [6,8,24]. In an effort to better understand the health effects of BPAF to humans, this study was undertaken to clarify the biotransformation of BPAF under physiological conditions. Four metabolites, including diglucuronide conjugated (M1), glucuronide conjugated (M2), glucuronide dehydrated (M3) and sulfate conjugated (M4), were identified in the urine of SD rats. M1, M2 and M3 were related to glucuronidation indicating that glucuronidation is an important reaction for BPAF metabolism. Among the four metabolites, BPAF-G was the only metabolite detected in the plasma of SD rats administrated with a single dose of BPAF, implying that M1 and M3 may be the byproducts or reactive intermediates produced during BPAF glucuronidation. Sulfate conjugation (M4) is also detected, suggesting that there are other metabolism pathways and different enzymes responsible for BPAF biotransformation *in vivo*. However, the value of mass spectrometry response for M4 was substantially lower than that for M2, which suggests that sulfate conjugation is not likely the major pathway for BPAF biotransformation *in vivo*. 

Chemicals conjugated with glucuronic acid or sulfonate, which are known as Phase II reactions, tend to be less active than their parent chemicals. Biotransformation of BPAF to glucuronide and sulfate conjugation indicated that metabolic Phase II conjugation of BPAF does occur *in vivo*. Several studies on the biotransformation of BPA have been carried out both *in vivo* and *in vitro* [15,17,25-27]. BPA glucuronide was characterized as the major metabolite of BPA, while other metabolites such as BPA diglucuronide, BPA sulfate conjugate, and 5-hydroxybisphenol A were identified as well [28]. Based on previous studies and our results, both BPA and BPAF have the metabolites with glucuronide, diglucuronide and sulfate conjugation, which suggests that BPAF has similar metabolic pathway to BPA *in vivo*. 

EDCs have been extensively studied for their potential adverse health effects, including infertility, birth defects, development and progression of breast cancer and islet disruption [29-33]. The rate of BPAF glucuronidation has implications for predicting the potential threat of BPAF in humans. According to our study, biotransformation of BPAF occurred quickly *in vivo*, which resulted in the lower level of BPAF compared to BPAF-G in plasma. Therefore, additional experiments are necessary to explore the toxicology of BPAF-G, which may reflect a more authentic burden on BPAF exposure.

UGT family plays an important role in the chemical reaction of glucuronidation [21]. It has been reported that UGT2B1 is mainly responsible for glucuronidation of BPA in rat liver microsomes [14]. Human UGT2B7 shares similar sequence homology to rat UGT2B1. Therefore, we speculated that BPAF could be metabolized to BPAF-G by human UGT2B7. Our results showed that human UGT2B7 has the highest efficiency for BPAF glucuronidation among the 12 human UGTs tested. This result suggests that the effects of BPAF in human beings may depend on the presence or absence of the UGT isoforms corresponding to UGT2B7 expressed in the liver, kidney and small intestine [34], and that overexpression of UGT2B7 could decrease the BPAF burden in humans. Expression of UGT2B7 in fetal liver is much lower than that in the adults [35], which suggests that the threat of BPAF to fetus can be more severe than that to adults, because BPAF is unable to be metabolized in the fetus following the maternal exposure. On the other hand, the glucuronidation rate of UGT2B7 is markedly lower than that of HLM, suggesting other UGT isoforms with higher efficiency of BPAF glucuronidation than UGT2B7 could exist in HLM. 

Metabolism could play an essential role in modulating the estrogenic activities of EDCs [17,36]. It has been reported that metabolism of BPA to BPA glucuronide will decrease BPA-induced estrogenic potency [28]. According to our results, BPAF-G did not show any estrogenic activation compared to BPAF, indicating that BPAF could be metabolized to the inactive form, which is similar to BPA. These results suggest that glucuronidation by UGT family *in vivo* may serve as a general defensive mechanism against BPAF and other EDCs. 

Chronic exposure to multiple EDCs is considered as a cause of increasing the risk of developing breast cancer [37-39]. Experimental studies showed that BPA could stimulate carcinogenesis [39,40] and promote the proliferation of cancer cells by activating the ER [41,42]. BPA can also induce tumor aggressiveness in clinical tissue of breast cancer patients [43]. Since BPAF exhibits more estrogenic potency than BPA [4,44], BPAF should pose more severe threats to human beings, especially to hormone-sensitive tumor patients. According to our study, biotransformation of BPAF to BPAF-G could be a general mechanism to decrease BPAF-induced threat to humans. 

In the present study, we identified four metabolites of BPAF including BPAF diglucuronide, BPAF glucuronide (BPAF-G), BPAF glucuronide dehydrated and BPAF sulfate in the urine of SD rats. After determination of BPAF metabolites in the plasma of SD rats, we found that BPAF glucuronidation is a rapid and efficient pathway for the biotransformation of BPAF, and BPAF-G was considered as a major metabolite *in vivo*. In addition, biotransformation of BPAF to BPAF-G was also confirmed in the incubation with HLM as well as UGTs, including UGT1A1, UGT1A3, UGT1A8, UGT1A9, UGT2B4, UGT2B7, UGT2B15 and UGT2B17 *in vitro*. In ER-positive breast cancer T47D and MCF7 cells, ER-regulated gene expression and cell proliferation were stimulated by BPAF, not by its metabolite BPAF-G. These results clearly show that biotransformation of BPAF to BPAF-G could eliminate BPAF-induced estrogenic activity, implying that biotransformation of BPAF has a key biological significance to decrease the potential risk to humans, especially to estrogen-sensitive cancer patients. Our findings thus provide a possible defensive mechanism against BPAF *in vivo*. 

## Supporting Information

File S1 **Figure S1, NMR spectrum of BPAF-G. Figure S2, Identification of BPAF metabolites in the incubation with HLM and UGT reaction mixture. Table S1, Method validation for BPAF and BPAF-G. Table S2, NMR data of BPAF-G (DMSO-d6). Method S1, Isolation and purification of BPAF metabolites. Method S2, UPLC conditions for UPLC/ESI-QTOF-MS analysis. Method S3, Method validation for BPAF and BPAF-G.**
(DOC)
