# Supplementary material for: Biotransformation of Bisphenol AF to Its Major Glucuronide Metabolite Reduces Estrogenic Activity
Source: PLoS One. 2013 Dec 13;8(12):e83170. doi: 10.1371/journal.pone.0083170 (PMC3862725; doi:10.1371/journal.pone.0083170)
Supplement: File S1 — Figure S1, NMR spectrum of BPAF-G. Figure S2, Identification of BPAF metabolites in the incubation with HLM and UGT reaction mixture. Table S1, Method validation for BPAF and BPAF-G. Table S2, NMR data of BPAF-G (DMSO-d6). Method S1, Isolation and purification of BPAF metabolites. Method S2, UPLC conditions for UPLC/ESI-QTOF-MS analysis. Method S3, Method validation for BPAF and BPAF-G. (DOC) [file pone.0083170.s001.doc]

**Supporting Informaiton**

**Figure S1**

**
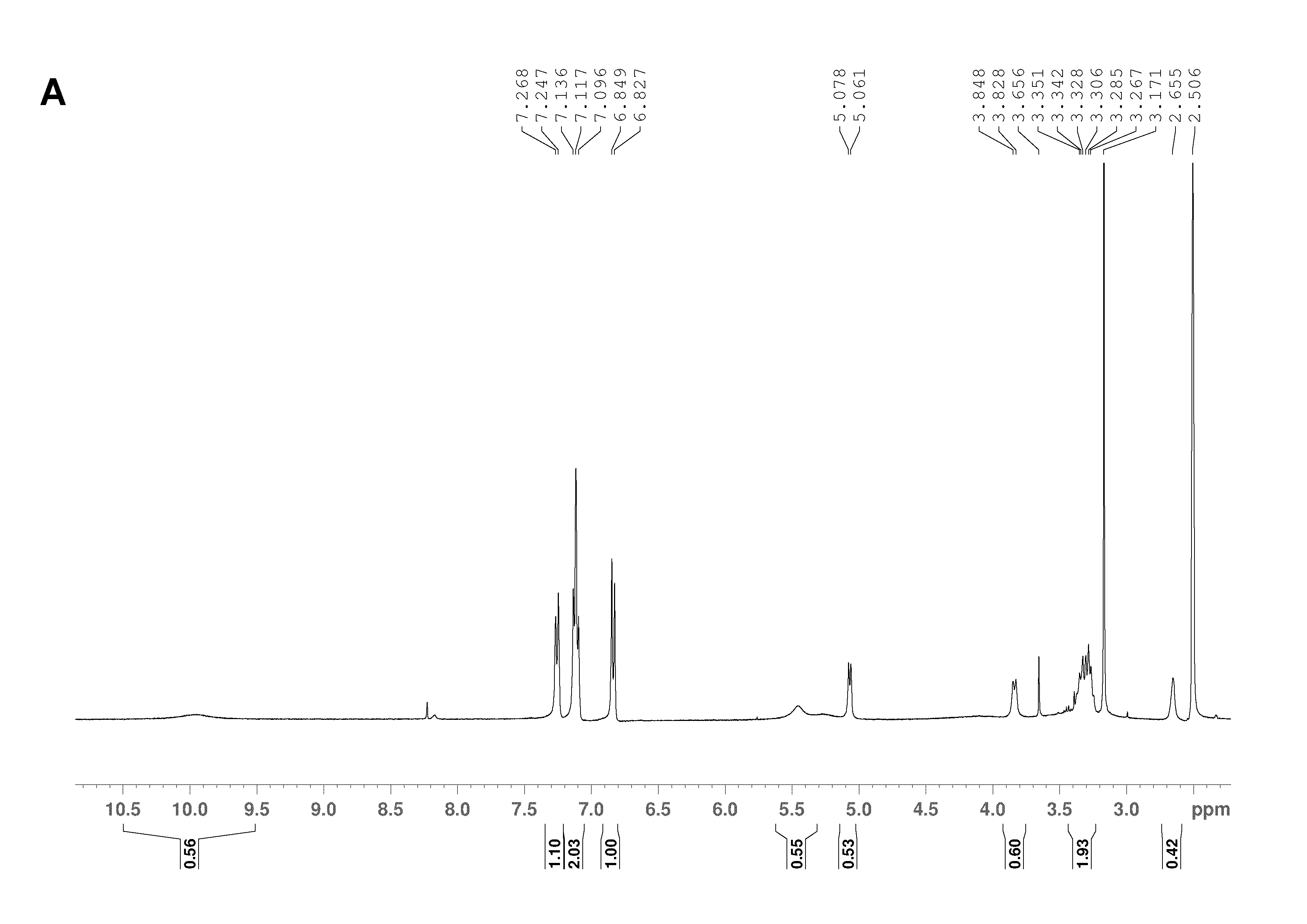
**

**
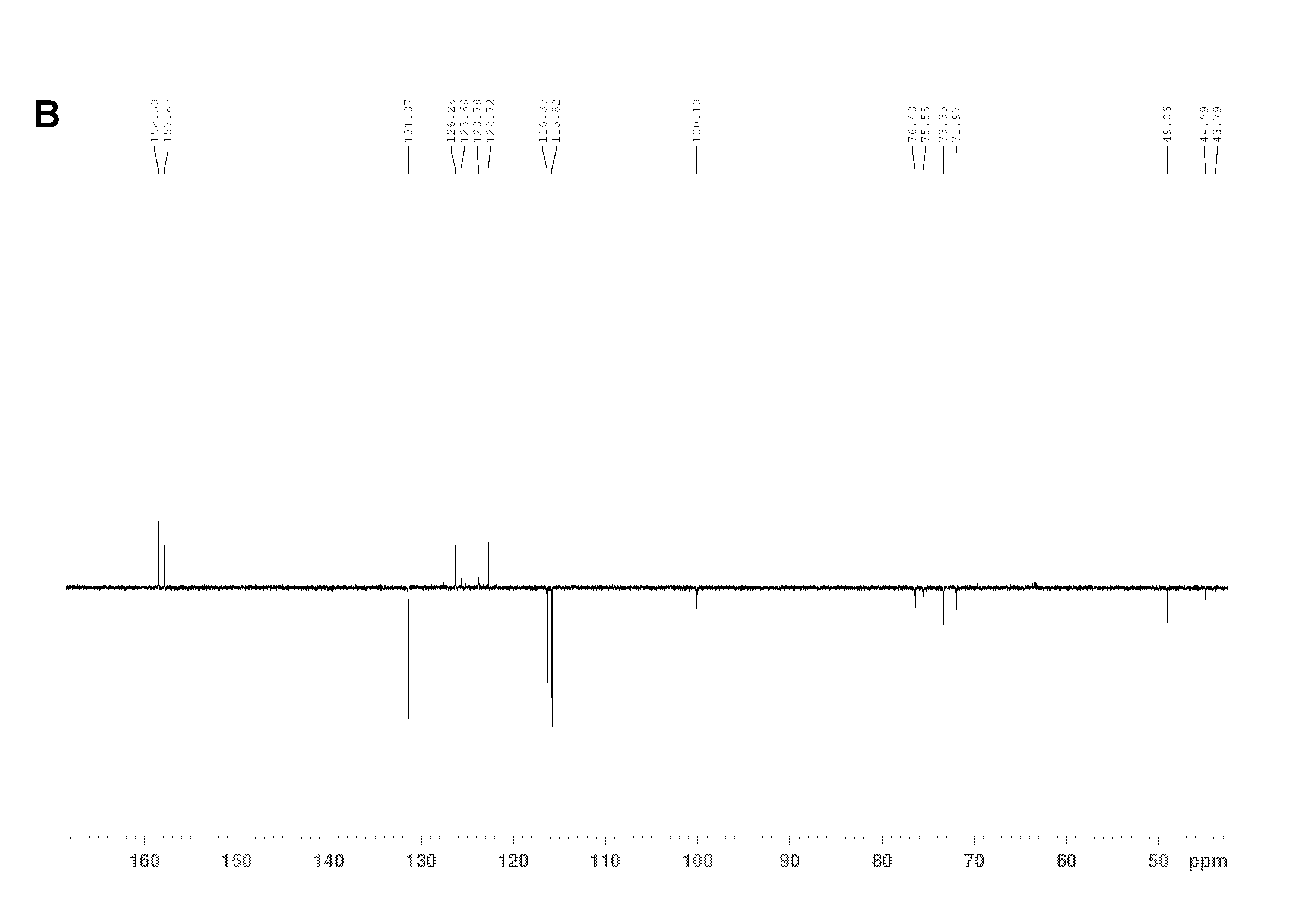
**

**Figure S1 NMR spectrum of BPAF-G.** (A) ^1^H NMR spectrum of BPAF-G. (B) ^13^C NMR spectrum of BPAF-G. ^1^H and ^13^C NMR spectra were performed on a Bruker AV 400 spectrometer. ^1^H and ^13^C NMR were tested in DMSO-d_6_. Chemical shifts were given on the δ-scale relative to TMS.

**Figure S2**

**
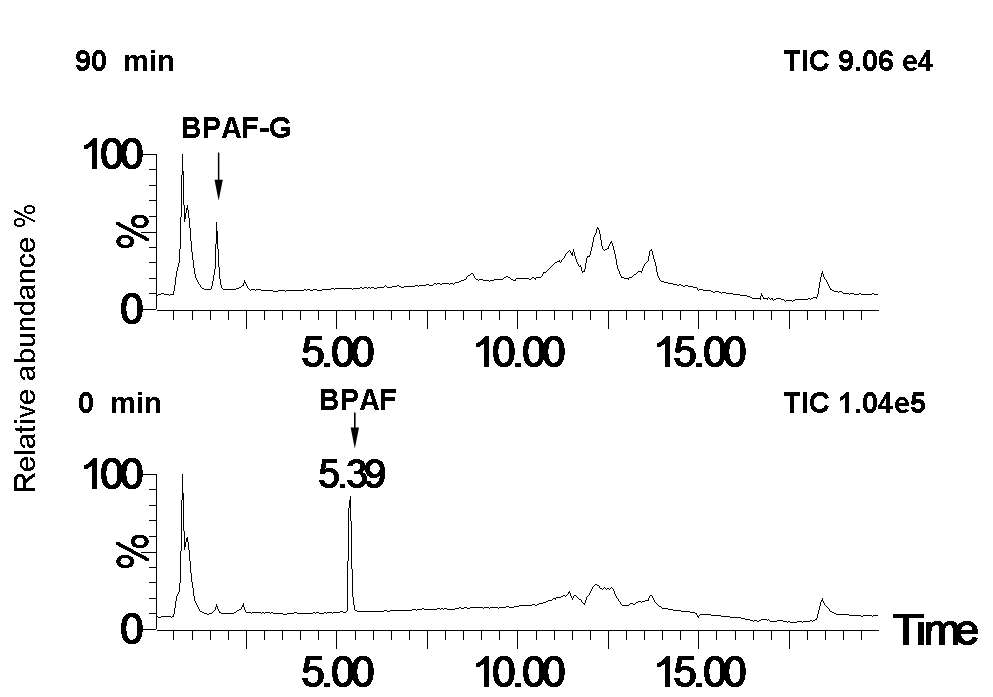
**

**Figure S2 Identification of BPAF metabolites in the incubation with HLM and UGT reaction mixture.** BPAF (10 µM) was incubated with HLM (100 µg/mL) in the presence of UGT reaction buffer. The samples were collected at 90 min. BPAF-G was the only metabolite detected by UPLC/ESI-QTOF-MS.

**Table S1 Method validation for BPAF and BPAF-G**.

|  | Spiking level (µg/L) | Recovery (%) | RSD  (%, *n* = 5) | MLOD/MLOQ(µg/L) |
| --- | --- | --- | --- | --- |
| BPAF | 1 | 106.7 | 10.89 | 0.5/1 |
|  | 2 | 92.4 | 3.09 |  |
|  | 20 | 84.5 | 1.35 |  |
| BPAF-G | 10 | 89.1 | 3.96 | 3/10 |
|  | 20 | 82.8 | 8.54 |  |
|  | 400 | 97.6 | 2.55 |  |

**Table S2** NMR data of BPAF-G (DMSO-d6).

|  | ^1^H NMR  δ | ^13^C NMR  δ |
| --- | --- | --- |
| 1 |  | 126.3 |
| 2,6 | 7.12 | 131.4 |
| 3,5 | 7.25 | 115.8 |
| 4 |  | 157.8 |
| 7 |  | 63.3 |
| 8,9 |  | 122.7 |
| 1′ |  | 126.3 |
| 2′,6′ | 7.10 | 131.4 |
| 3′,5′ | 6.83 | 116.4 |
| 4′ |  | 158.5 |
| 1″ | 5.06 | 100.1 |
| 2″ |  | 73.4 |
| 3″ |  | 75.6 |
| 4″ |  | 72.0 |
| 5″ | 3.83 | 76.4 |
| 6″ |  | 171.0 |

**
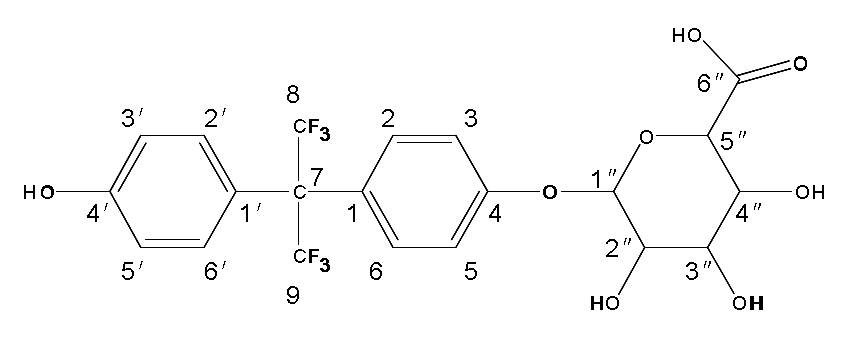
**

**Method S1 Isolation and purification of BPAF metabolites.**

Pooled urine samples (200 mL) were added to 100 mL cartridges filled with Supelco Discovery® DSC-18 SPE Bulk Packing (3,000 mg, 50 µm, Bellefonte, PA, USA) for the initial purification. After sample loading, 30 mL of a 50% MeOH/H_2_O solution was used to eliminate interference, and the metabolites were eluted with a 10 mL 70% MeOH/H_2_O solution. The collected fraction (10 mL) was pooled and dried to 2 mL under a stream of nitrogen at 40 ºC for further purification, then separated by semi-preparative HPLC.

Semi-preparative HPLC was performed on an Alltech 426 apparatus（Deerfield, Illinois, USA）with a 3300-ELSD UV detector. Separations were carried out with a Venusil XBP C18 column (10×250 mm, 5 µm) from Agela Technologies (Newark, DE, USA). The mobile phase was a mixture of MeOH and water (55/45, v/v) at a stable flow rate of 2.0 mL/min. The eluent was monitored with a UV detector at 226 nm. The BPAF-G was collected at its retention time and dried under a stream of air at 50 ºC before it was structurally confirmed by NMR.

**Method S2 UPLC conditions for UPLC/ESI-QTOF-MS analysis.**

UPLC working conditions for UPLC/ESI-QTOF-MS analysis was conducted as follows: the column oven was set to 40 ºC, the injection volume was 5 μL, and the flow rate was 0.3 mL/min. The mobile phase was composed of solvents A (MeOH) and B (0.1% ammonia in water). For urine samples, the gradient elution began with 10% A, raised to 80% A in 20 min, then raised to 100% A in 24 min, kept at 100% A for 2 minutes and finally lowered back to 10% A for 4 min. For the samples collected form the *in vitro* reaction mixture, a flow rate of 0.3 mL/min was used as a gradient elution starting with 50% A, followed by a 15 min linear gradient to 100% A and held for 2.0 min, finally returned to the initial state to equilibrate for 3 min before the next injection.

**Method S3 Method validation for BPAF and BPAF-G.**

Recovery was evaluated using 100 µL samples spiked with 1, 2 and 20 µg/L of BPAF; 10, 20 and 400 µg/L BPAF-G. Precision, expressed as percent relative standard deviation (RSD %), was determined by five replicates of the biological matrices spiked with the metabolite. The method limit of detection (MLOD) and the method limit of quantization (MLOQ) were defined as the minimum detectable amount of analyte from samples in MRM mode with signal-to-noise ratios of 3:1 and 10:1, respectively. Matrix effects were defined by subtracting the ratio between the slope of matrix-matched standard curves and the slope of standard solution curves, then multiplying by 100 to obtain a percentage. The signal was enhanced if the value was negative and suppressed if the value was positive.

The results showed that the correlation coefficients (*r*^2^) of the matrix-fortified calibration curves were all greater than 0.99. The recovery at three fortified levels ranged from 82.8% to 106.7%, with the RSD lower than 10.9% (Table S1). The MLOD of BPAF and BPAF-G were 0.5 µg/L and 3 µg/L, respectively. The MLOQ of BPAF and BPAF-G were 1 µg/L and 10 µg/L, respectively. Matrix effects calculated in plasma for BPAF and BPAF-G were both lower than 5%.
